# Socioeconomic dynamics and tuberculosis mortality in São Paulo: a population-level analysis using spatial modeling

**DOI:** 10.1016/j.lana.2026.101572

**Published:** 2026-07-13

**Authors:** Yan Mathias Alves, Reginaldo Bazon Vaz Tavares, Nathalia Zini, Tatiana Pestana Barbosa, Maria Eduarda Pagano Pelodan, Antônio Carlos Vieira Ramos, Tatiana Ferraz de Araújo Alecrim, Jaqueline Garcia de Almeida Ballestero, Bruno de Bezerril Andrade, Jason Edward Farley, Ricardo Alexandre Arcêncio

**Affiliations:** a Department of Maternal-Infant and Public Health Nursing, Ribeirão Preto College of Nursing, University of São Paulo, Ribeirão Preto, Brazil; b Department of Nursing, State University of Minas Gerais, Passos Câmpus, Passos, Minas Gerais, Brazil; c Division of Infectious Diseases, Department of Medicine, Johns Hopkins University, Baltimore, MD, United States of America; d Department of International Health, Bloomberg School of Public Health, Johns Hopkins University, Baltimore, MD, United States of America; e Clinical and Translational Research Laboratory, Gonçalo Moniz Institute, Oswaldo Cruz Foundation, Salvador, Bahia, Brazil; f Johns Hopkins University School of Nursing, Baltimore, MD, United States of America

**Keywords:** Tuberculosis mortality, Social determinants of health, Spatial-temporal modeling, Primary healthcare

## Abstract

**Background:**

Tuberculosis remains a major cause of preventable mortality in Brazil, marked by pronounced social and territorial inequalities. Evidence remains limited on how socioeconomic conditions and health system characteristics are associated with tuberculosis mortality across age groups in high-burden settings such as São Paulo state. This study aimed to examine the spatial and temporal associations of these factors with tuberculosis mortality across municipalities in the State of São Paulo, Brazil.

**Methods:**

We conducted a population-based ecological study in São Paulo, Brazil. All TB deaths reported to the Mortality Information System from 2020 to 2024 were included. Socioeconomic, demographic and health system factors were selected based on a conceptual framework. Variables associated with TB mortality were assessed using Generalized Additive Models for Location, Scale, and Shape, with spatial smoothing.

**Findings:**

The analysis included 38,700 municipality-month observations. In the final spatial GAMLSS model, proportions of household crowding (>2 residents/bedroom) and elderly population (>59 years) were associated with 3.25% and 5.92% increases in expected TB mortality for each one percentage-point increase, respectively. In contrast, each one percentage-point increase in primary health care coverage was associated with a 0.30% decrease in expected TB mortality. The fitted spatial effect indicated higher expected TB mortality along coastal and central regions, and lower expected mortality in the northwestern region.

**Interpretation:**

Tuberculosis mortality in São Paulo is shaped by persistent socioeconomic and territorial inequalities, with distinct patterns across age groups. These findings highlight the need for strategies that address structural vulnerability and strengthen primary health care to reduce avoidable tuberculosis-related deaths.

**Funding:**

This work was supported by Coordenação de Aperfeiçoamento de Pessoal de Nível Superior–Brasil and São Paulo State Research Foundation (FAPESP).

**Research in context**

**Evidence before this study**

Before undertaking this study, we searched PubMed/MEDLINE, Scopus, Web of Science, Embase, and SciELO for population-based and ecological studies assessing tuberculosis mortality and its association with socioeconomic conditions, demographic characteristics, health-system indicators, and territorial inequalities. Searches were conducted between January 10 and February 15, 2025, without language restrictions, and included studies published up to December 31, 2024. The following terms and their synonyms were used in different combinations: “tuberculosis mortality”, “tuberculosis deaths”, “social determinants”, “socioeconomic factors”, “health inequalities”, “spatial analysis”, “ecological study”, “longitudinal analysis”, “municipality”, “State of São Paulo” and “Brazil”. We also screened reference lists of eligible studies, national epidemiological reports, and documents from World Health Organization.

Available evidence consistently shows that tuberculosis mortality is strongly associated with poverty, social vulnerability, unequal access to healthcare, and regional disparities, particularly in high-burden settings such as Brazil. Previous studies have reported associations between mortality and indicators such as income inequality, primary healthcare coverage, urbanization, and access to health service. However, most investigations were based on cross-sectional or purely ecological approaches, frequently focused primarily on tuberculosis incidence rather than mortality outcomes, and have applied conventional regression methods with limited ability to address overdispersion, excess zeros, longitudinal dependence, and spatial heterogeneity in municipality-level data. Evidence integrating socioeconomic, demographic, healthcare, and spatiotemporal dimensions of tuberculosis mortality through flexible distributional modelling approaches remains scarce, particularly in large population-based analyses conducted in Brazil.

**Added value of this study**

This study provides a comprehensive population-level assessment of tuberculosis mortality across all municipalities in the State of São Paulo over a five-year period using generalized additive models for location, scale, and shape (GAMLSS) with spatial smoothing. By integrating demographic, socioeconomic, epidemiological, and healthcare indicators within a unified spatiotemporal analytical framework, the study enables the evaluation of complex and spatially heterogeneous associations. The findings show that household overcrowding, population ageing, higher tuberculosis incidence, and a greater proportion of Black and Brown population were associated with increased tuberculosis mortality, whereas higher primary healthcare coverage and improved municipal socioeconomic development were associated with lower mortality. The fitted spatial effect further identified territorial clusters with persistently elevated expected mortality, even after adjustment for measured covariates.

**Implications of all the available evidence**

Taken together, these findings reinforce that tuberculosis mortality is strongly shaped by persistent structural and territorial inequalities that cannot be addressed through biomedical interventions alone. Strengthening primary healthcare, improving living conditions, reducing racial and territorial inequities, and ensuring continuity of care are essential to reduce avoidable tuberculosis deaths in high-burden settings. At the policy level, the results support prioritizing socially vulnerable territories and integrating health and social policies to mitigate structural determinants associated with tuberculosis mortality. Future longitudinal studies incorporating individual-level data and explicit spatiotemporal approaches are needed to further clarify causal pathways and support more equitable tuberculosis control strategies.

## Introduction

Tuberculosis (TB) remains the leading cause of death from a single infectious agent worldwide, surpassing HIV/AIDS and ranking among the ten leading causes of global mortality.^1^ Despite being a preventable and curable disease, TB continues to reflect profound inequalities in access to health care and socioeconomic conditions. In 2023, an estimated 1.3 million people died from TB and 10.6 million became ill, highlighting persistent gaps in prevention and care efforts, particularly in low- and middle-income countries.^1^

Several studies have been conducted in Brazil to characterize the epidemiological profile of deaths attributable to TB.^2^, ^3^, ^4^, ^5^

Moreover, existing studies have largely focused on individual-level risk factors or specific regions, with limited attention to how socioeconomic conditions and health system characteristics interact across space and time at the population level.^3^^,^^5^ This represents a critical gap, particularly in highly unequal settings where structural determinants may shape distinct spatial patterns of TB mortality.

Population-level epidemiology recognizes that, although individual longitudinal data are ideal, valuable insights can still be derived from aggregated spatio-temporal analyses that capture social trajectories and health inequalities relevant to TB mortality. The use of advanced modeling techniques like Generalized Additive Models for Location, Scale, and Shape (GAMLSS) captures complex distributions and nonlinear effects of predictors over space and time.^6^^,^^7^

This approach strengthens understanding of how socioeconomic and environmental exposures interact with geographical contexts to influence TB susceptibility and illness over time, highlighting the persistent social inequalities underlying mortality from a preventable and curable disease. In this context, the state of São Paulo provides a particularly relevant setting for investigation. As the most populous and economically developed state in Brazil, it combines high TB burden with marked social and territorial inequalities, making it a critical context for understanding how structural determinants influence mortality patterns.^5^

Understanding these dynamics is essential to inform more targeted, equitable, and context-specific public health strategies aimed at reducing avoidable TB deaths and advancing progress toward TB elimination goals.

This study aims to examine how temporal dynamics, socioeconomic conditions, and health system characteristics spatially influence tuberculosis mortality across municipalities in the State of São Paulo.

## Methods

### Study design

This is a population-based ecological study,^8^ investigating the association between demographic, epidemiological, socioeconomic, and health services variables and TB mortality in the state of São Paulo, Brazil. Data were aggregated at the municipal level with monthly temporal resolution, covering the period from January 2020 to December 2024. This report adhered to the Strengthening the Reporting of Observational Studies in Epidemiology (STROBE) reporting guidelines.^9^

### Study setting

The study was conducted in the state of São Paulo, located in southeastern Brazil. São Paulo is the most populous state in the country, comprising 645 municipalities, with an estimated population of 44,411,238 in 2022 and a population density of 178.92 inhabitants per km^2^.^10^

### Participants, variables and data sources

The outcome of interest in this study was the number of TB deaths. These data were obtained from Mortality Information System (Sistema de Informações sobre Mortalidade, SIM) in which the death was reported in any municipality of São Paulo and between January 2020 and December 2024. TB deaths were defined as deaths for which any form of TB was recorded as the underlying cause of death by a physician on the death certificate, according to ICD-10 codes A15.0–A19.9. According to the Brazilian Ministry of Health, a TB death occurs when the disease initiates the chain of pathological events that directly lead to death.^11^ Given that the registries on TB mortality was obtained considering the International Classification of Diseases (ICD-10) codes ICD-10 codes A15–A19. The municipality of occurrence and the date of death recorded in SIM were used to define the geographical and temporal references of the outcome.

The candidate explanatory variables were selected based on the literature^5^^,^^12^, ^13^, ^14^, ^15^, ^16^, ^17^, ^18^ and were informed by a conceptual framework grounded in the determinants of TB in the Brazilian context.^16^ Because this was an ecological study, the selected variables were mainly aligned with the social and contextual vulnerability axis of the framework. This axis includes factors related to poverty, overcrowded housing, lack of social protection, community TB prevalence, high population density, and geographic, economic, and cultural barriers.^16^

The selected variables were organized into four conceptual domains: (1) Socioeconomic: which included the municipal development index (composed of income, education and longevity), proportion of households receiving cash transfer program benefits, and proportion of residents aged 18 years or older with no schooling or incomplete elementary education; (2) Demographics: which included population density per km^2^, proportion of residents aged 60 years or older and proportion of Black and mixed population; proportion of households with more than 2 residents per bedroom, proportion of households with inadequate sewage disposal; (3) Healthcare: which included primary healthcare coverage and rates of nurses and physicians, and per 1000 inhabitants; (4) Epidemiological: which included monthly TB incidence rate per 100,000 inhabitants, monthly COVID-19 incidence rate per 100,000 inhabitants; and rate of population deprived of liberty per 100,000 inhabitants.

This data was obtained from official secondary data sources. For each variable, the data source, reference year or period and calculation procedure are described in Table S1. TB incidence rates were calculated using the number of reported TB cases from the database provided by the State Center for Epidemiological Surveillance “Prof. Alexandre Vranjac”. These data were the only non-publicly available data used in the study and were obtained in November 2025, without patient identifiers.

Municipal population data from the 2022 Brazilian Demographic Census, provided by the Brazilian Institute of Geography and Statistics, were used as the population denominator. In the regression models, the logarithm of the 2022 municipal population was included as an offset term to account for differences in population size across municipalities and to model TB deaths as mortality rates rather than absolute counts.

COVID-19 incidence was evaluated as a covariate to account for the potential relationship between local pandemic burden and monthly TB mortality, considering previous evidence on the impact of the COVID-19 pandemic on TB services, notifications, and mortality.^2^^,^^18^ Because the municipal COVID-19 incidence series started in March 2020, this assessment was conducted in the subset of months with available COVID-19 data. However, COVID-19 incidence was not retained in the GAIC-based stepwise selection procedure and was therefore not included in the final model.

### Missing data and bias

Some municipal-level covariates were not available monthly or for all years of the study period. Therefore, values were temporally harmonized to the monthly municipal panel according to the reference period of each data source, as described in Supplementary Methods (Table S1). The analytical panel included 38,700 municipal-month observations, corresponding to 645 municipalities observed over 60 months. The municipal development index was available for 2020 and 2022; therefore, 2020 values were assigned to 2020 and 2021, and 2022 values were assigned to 2022, 2023, and 2024. Thus, 23,220 municipal-month observations (60.0%) were assigned from the nearest available index reference year outside the year of direct measurement. The proportion of residents aged 18 years or older with no schooling or incomplete elementary education, population density, proportion of households with more than 2 residents per bedroom, proportion of households with inadequate sewage disposal and the proportion of residents aged 60 years or older were obtained from the 2022 Brazilian Demographic Census and were treated as fixed municipal-level reference covariates for the entire study period. For these census-derived variables, 30,960 municipal-month observations (80.0%) corresponded to months outside the 2022 reference year.

Population density and the proportion of residents aged 60 years or older were available annually and were assigned to the corresponding months of each year. The rate of the population deprived of liberty was obtained from the National Penitentiary Department Information System, which reports data by semester; therefore, first-semester values were assigned to January through June and second-semester values to July through December of each year. Because semester-specific values were available for the study period, no semester was missing, although values were harmonized to monthly observations within each semester. This approach was adopted because these variables were derived from census or periodically reported administrative sources, for which monthly measurements were not available. This approach is further addressed in the limitations section.

To reduce outcome ascertainment bias, all TB-related deaths recorded in SIM during the study period were included and classified using standardized ICD-10 codes for TB as the underlying cause of death, rather than relying exclusively on TB surveillance or treatment notification systems. To avoid purely data-driven covariate selection, candidate variables were first defined according to theory-informed conceptual domains and then evaluated using epidemiological plausibility, residual diagnostics and interpretability. Overall temporal variation was addressed by including a sequential monthly time variable, and residual temporal autocorrelation was assessed with Ljung–Box test. Spatial dependence was assessed using Global Moran's I,^19^ and a two-dimensional spatial smoothing term was included to reduce residual spatial dependence not explained by observed covariates.

### Statistical analysis

#### Regression modeling

Associations between monthly TB deaths and municipal-level covariates were assessed using Generalized Additive Models for Location, Scale and Shape (GAMLSS).^6^ This framework was selected because it allows flexible modelling of the response distribution and, when appropriate, its parameters for location, scale, skewness, and kurtosis using parametric and non-parametric functions of covariates^6^^,^^20^ This flexibility was relevant for the present study because the outcome consisted of monthly municipal counts of TB deaths across 645 municipalities. These data were characterized by many zero counts, overdispersion, and occasional high values in larger or high-burden municipalities. GAMLSS was therefore used as a flexible distributional regression framework that allowed the comparison of alternative count distributions and the incorporation of parametric terms and smooth functions.

Following the formulation proposed by Rigby and Stasinopoulos (2005),^6^ the GAMLSS framework can be expressed, for each distribution parameter *theta_k*, as:

$$g_{k}\left(\theta_{k}\right)\;=\;\eta_{k}\;=\;X_{k}\beta_{k}\;+\;\sum_{j\;=\;1}^{jk}h_{jk}\left(X_{jk}\right)$$

where *g*_*k*_*(.)* is a link function, *X*_*k*_*β*_*k*_ represents the parametric component, and *hⱼ*_*k*_ *(xⱼ*_*k*_*)* represents smooth non-parametric functions of covariates. In this study, the main model was specified for the location parameter of the distribution, with the monthly number of TB deaths as the outcome and the natural logarithm of the municipal population included as an offset.

Candidate count distributions were compared to identify the most appropriate specification for the outcome. Poisson, negative binomial type I, negative binomial type II, Poisson-inverse Gaussian, zero-inflated Poisson, zero-inflated Poisson type II, and zero-inflated negative binomial distributions were evaluated using the generalized Akaike information criterion (GAIC), Bayesian information criterion/Schwarz Bayesian criterion (BIC/SBC), convergence, and residual diagnostics.^7^ The negative binomial type I distribution was selected for the final model because it provided the best overall balance between model fit, convergence, and residual behavior. Diagnostic plots of randomized quantile residuals indicated an overall acceptable fit, although some deviation was observed in the upper tail, which did not improve significantly with other distributions.

After conceptual selection of candidate covariates, they were evaluated in the GAMLSS models considering epidemiological interpretability, coefficient stability and information criteria. A GAIC-based stepwise procedure, consistent with GAMLSS model-building strategies, was used as a complementary tool to compare candidate specifications.^20^ Multicollinearity was assessed using correlation matrices and variance inflation factors. Variance Inflation Factor values were interpreted as diagnostics of potential coefficient instability, no exclusion thresholds were used.

TB mortality is known to be spatially heterogeneous and may reflect unmeasured territorial processes.^21^ Therefore, spatial structure was incorporated into the GAMLSS model following the spatial modelling framework described by De Bastiani et al. (2018),^20^ in which spatial components can be included within the distributional regression structure. In the final model, residual spatial heterogeneity was represented by a two-dimensional thin-plate regression spline fitted to the municipal centroid coordinates. This coordinate-based smooth surface was used to capture broad spatial variation across the 645 municipalities that was not explained by the selected covariates. The basis dimension of the spatial smooth was defined by comparing alternative specifications. The adopted model was assessed based on model fit and evidence of no residual spatial autocorrelation, evaluated using Global Moran's I.^19^^,^^22^

An overall temporal component was included in the model to account for underlying temporal variation in monthly TB mortality over the study period, which covered the COVID-19 pandemic and post-pandemic years.^2^^,^^18^ Alternative temporal specifications were evaluated, including no temporal term, a linear monthly trend, a penalized spline smooth of time, month-of-year fixed effects, and a combination of smooth temporal trend and month-of-year effects. These specifications were compared using GAIC and the linear monthly trend was retained in the final model. Residual temporal autocorrelation was assessed using autocorrelation function plots and Ljung–Box tests applied to monthly aggregated mean residuals.

### Descriptive analysis

Descriptive analyses were conducted for the candidate explanatory variables using frequency tables, histograms, and measures of central tendency and dispersion.

### Spatial analysis

Choropleth maps were constructed to describe the spatial distribution of the mean monthly TB mortality rate and the covariates included in the final model. For variables measured monthly, municipal mean values over the study period were mapped. For variables derived from census or other reference-year sources, values were mapped according to their original reference period.

Global Moran's I was used to assess spatial autocorrelation in the outcome and final model covariates. This analysis was conducted to identify whether the spatial distribution of each variable showed evidence of clustering across municipalities.

All analyses were conducted in the R programming language using RStudio software, version 4.2.1 (Posit Software, PBC, Boston, USA). Data management and visualization were performed using tidyverse packages. Spatial data processing, mapping, and spatial autocorrelation analyses were conducted using sf and spdep. GAMLSS models were fitted using gamlss, gamlss.dist, and gamlss.add, with two-dimensional spatial smoothing implemented through the ga () interface to smooth terms from the mgcv framework.

### Ethical aspects

The study was approved by the Research Ethics Committee of the Ribeirão Preto College of Nursing, University of São Paulo (EERP/USP), under Certificate of Ethical Appreciation (CAAE) No. 77580424.5.0000.5393, in accordance with Resolution No. 466/2012 of the Brazilian National Health Council.

### Role of the funding source

The funders had no role in the study design, data collection, data analysis, data interpretation, manuscript preparation, or the decision to submit the manuscript for publication. The corresponding author had full access to all study data and had final responsibility for the decision to submit the manuscript.

## Results

### Descriptive analysis

Between January 2020 and December 2024, 5896 TB deaths were recorded in São Paulo state. The mean monthly number of TB deaths was 98.3 (standard deviation [SD] 20.3), corresponding to a mean monthly mortality rate of 0.22 deaths (SD 0.05) per 100,000 population. The cumulative mortality rate over the study period was 13.28 deaths per 100,000 population, and the average annual mortality rate was 2.66 deaths (SD 0.43) per 100,000 population.

**Fig. 1:**
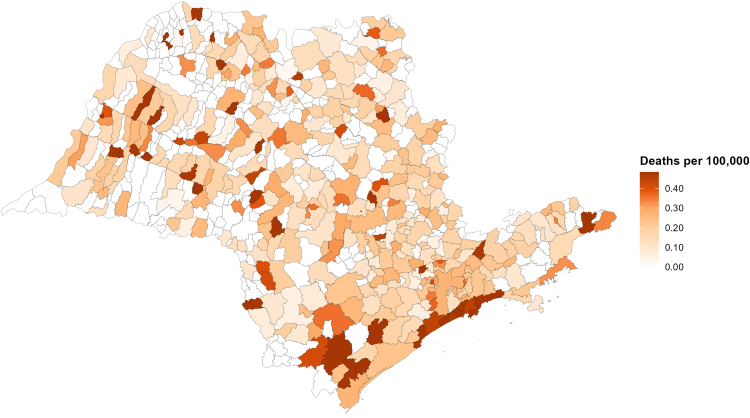
Spatial distribution of the mean monthly TB mortality rate, São Paulo, Brazil, 2020–2024.

Fig. 1 shows the mean monthly TB mortality rate per 100,000 population across municipalities of São Paulo state between 2020 and 2024. The Global Moran's I for the TB mortality was 0.1 with p-value <0.01, indicating a significant spatial autocorrelation.

Descriptive statistics for the outcome and candidate covariates are presented in Table 1. The monthly number of TB deaths had a median and interquartile range of 0, with a maximum value of 52, indicating a right-skewed distribution with a high frequency of zero counts.

**Table 1:** Descriptive statistics of the outcome and candidate covariates, São Paulo, Brazil, 2020–2024.

Variable name	Coded variable	Mean (SD)	Median (IQR)	Minimum	Maximum
Primary health care coverage (%)	cobertura_aps	89.21 (39.05)	96.05 (65.77–100)	0	427.66
Population density	dens_demograf	329.42 (1282.9)	40.15 (20.21–117.41)	3.5	13,465.62
COVID-19 incidence rate per 100,000 population	incid_covid	368.72 (1500.9)	50.4 (0–376,0)	0	96,300
TB incidence rate per 100,000 population	incid_tb_100mil	2.61 (7.65)	0 (0–2.65)	0	363.54
São Paulo Municipal Development Index	ipdm	0.55 (0.05)	0.55 (0.51–0.58)	0.39	0.71
Monthly TB deaths	obitos_tb	0.15 (1.36)	0 (0–0)	0	52
2022 Census Population (Offset)	pop_2022	68,854.63 (46,4066.8)	13,163 (5466–38,324)	907	11,451,999
Households receiving Bolsa Família benefits (%)	porcent_familia_benef_pbf_por_total_domicilio	15.45 (7.89)	13.68 (9.81–19.49)	1.76	60.76
Households with inadequate sewage disposal (%)	prop_esgotamento_inadequado	9.17 (10.01)	5.54 (2.63–11.44)	0	69.58
Households with more than 2 residents per bedroom (%)	prop_mais2_dorm	8.65 (3.35)	7.82 (6.23–10.39)	1.26	23.05
Population aged 60 years or older (%)	prop_mais59	17.97 (3.63)	17.82 (15.53–20.12)	7.67	33.85
Black or brown population (%)	prop_preta_parda	39.51 (9.47)	38.28 (32.51–46.51)	18.13	77.43
Adults without completed elementary education (%)	prop_sem_fundamental_18mais	34.3 (7,1)	35.17 (29.13–39.38)	13.72	59.2
Nurses per 1000 population	taxa_enf_1000	1.28 (0.62)	1.15 (0.87–1.55)	0	6.22
Physicians per 1000 population	taxa_med_1000	1.52 (1.29)	1.14 (0.69–1.96)	0	12.55
Incarceration rate per 100,000 population	taxa_pen_100mil	1385.47 (6201.49)	0 (0–0)	0	75,070.75

Note: SD, Standard deviation; IQR, Interquartile range; TB, Tuberculosis.

### Spatial analysis

**Fig. 2:**
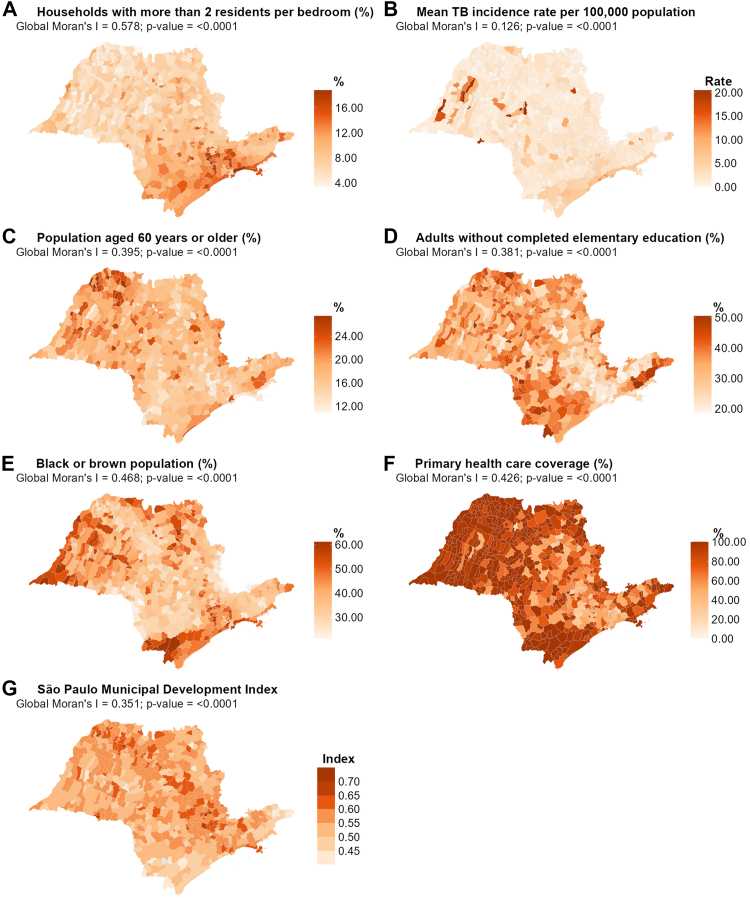
Spatial distribution of covariates included in the final GAMLSS model, São Paulo, Brazil, 2020–2024.

All covariates included in the final model showed statistically significant positive spatial autocorrelation according to Global Moran's I, indicating non-random spatial distribution across municipalities. Fig. 2 presents the spatial distribution of the covariates retained in the final GAMLSS model.

Spatial autocorrelation was strongest for households with more than two residents per bedroom (I = 0.578; p < 0.0001), proportion of Black or Brown population (I = 0.468; p < 0.0001), and primary healthcare coverage (I = 0.426; p < 0.0001). Mean TB incidence showed weaker but still significant spatial autocorrelation (I = 0.126; p < 0.0001).

### Final association model

Table 2 presents the final spatial GAMLSS model associated with the outcome at p-value <0.01.

**Table 2:** Parametric covariate estimates from the spatial GAMLSS model for monthly TB deaths in São Paulo, Brazil, 2020–2024.

Variable name	Coded variable	Estimate	Standard error	Exponential of the estimate	p-value
Monthly time trend	time_id	0.0048	0.0009	1.0048	<0.0001
Households with more than 2 residents per bedroom (%)	prop_mais2_dorm	0.0211	0.0073	1.0213	=0.0041
TB incidence rate per 100,000 population	incid_tb_100mil	0.0211	0.0014	1.0213	<0.0001
Population aged 60 years or older (%)	prop_mais59	0.0432	0.0077	1.0442	<0.0001
Adults without completed elementary education (%)	prop_sem_fundamental_18mais	−0.0324	0.0037	0.9681	<0.0001
Black or brown population (%)	prop_preta_parda	0.0142	0.0032	1.0143	<0.0001
Primary health care coverage (%)	cobertura_aps	−0.0025	0.0007	0.9975	=0.0002
São Paulo Municipal Development Index	ipdm	−3.7159	0.5513	0.0243	0.0001

Note: TB, tuberculosis; GAMLSS, Generalized Additive Model for Location, Scale and Shape.

The monthly temporal trend was positively associated with TB mortality. Each additional month was associated with a 0.48% [ (exp (0.0048) −1) ∗ 100] increase in the expected number of municipal-monthly TB deaths. Higher TB incidence was also associated with higher TB mortality, with each additional TB case per 100,000 population associated with a 2.13% increase in expected TB deaths.

Among the contextual variables, each one percentage-point increase in the proportion of households with more than two residents per bedroom was associated with a 2.13% increase in expected TB deaths. Similarly, each one percentage-point increase in the proportion of residents aged 60 years or older was associated with a 4.41% increase, and each one percentage-point increase in the proportion of Black or brown residents was associated with a 1,43% increase in expected TB deaths.

For variables inversely associated with TB mortality, higher primary health care coverage was associated with lower expected TB mortality. Each one percentage-point increase in coverage was associated with a 0.25% decrease in expected TB deaths. The proportion of adults without completed elementary education also showed an inverse association, with each one percentage-point increase associated with a 3.18% decrease in expected TB deaths.

**Fig. 3:**
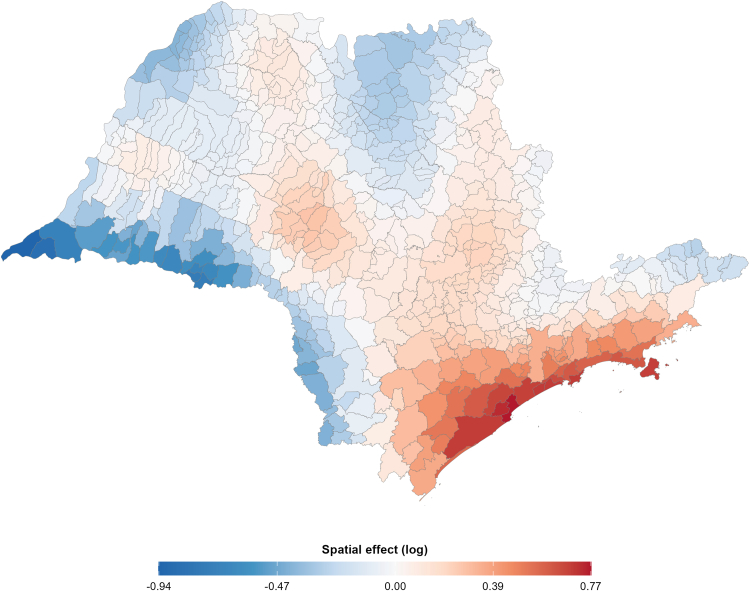
Fitted spatial effect for mu in the final spatial GAMLSS model. Note: log = logarithmic.

Fig. 3 shows the fitted spatial effect for the location parameter *mu* in the final spatial GAMLSS model. Positive values indicate municipalities with higher expected TB mortality, mainly along the coastal area and central regions of the state. Negative values indicate lower expected TB mortality, mainly in the northwestern region.

Relative to the reference spatial effect, the municipality with the highest fitted spatial effect had an expected TB mortality approximately 116.82% higher [(e0.77 − 1) ∗ 100], whereas the municipality with the lowest fitted spatial effect had an expected TB mortality approximately 60.8.8% lower [(1 − e−0.94) × 100%].

### Diagnostic plots

**Fig. 4:**
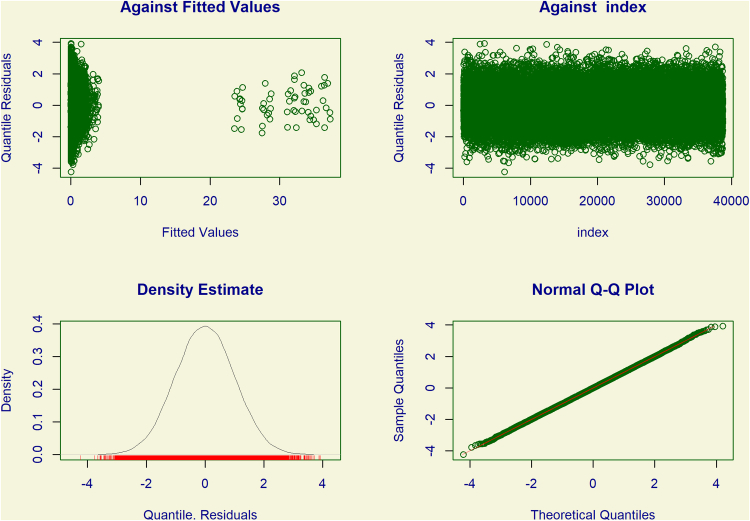
Residual plots from the final GAMLSS fitted model.

Model fit was assessed through quantile residual diagnostic plots (Fig. 4). The residuals showed an approximately normal distribution, as evidenced by the density curve and the Q–Q plot, in which the points closely follow the reference diagonal across almost the entire range. The absence of systematic trends in the residuals-versus-index plot indicates independence among observations. However, the residuals-versus-fitted-values plot revealed heterogeneity in residual dispersion at higher predicted values, suggesting limitations in model fit for that range of the response variable.

**Fig. 5:**
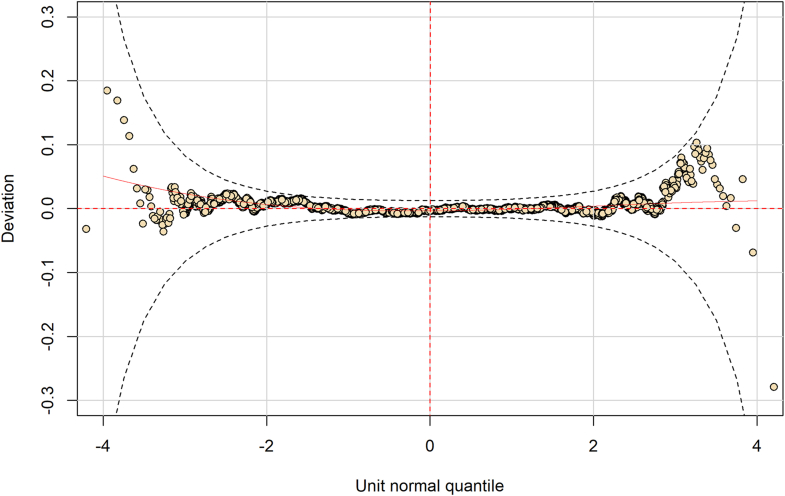
Worm plot of the final GAMLSS fitted model residuals.

The worm plot (Fig. 5) is a detrended normal Q–Q plot of the residuals, indicating a reasonable fit for the majority of the data, as most points lie within the 95% pointwise confidence bands. However, deviations were observed in the extreme tails.

For the sensitivity analysis, we removed each covariate that required greater temporal assignment separately from the final model and compared the reduced models with the final model. The final model had the lowest AIC, while the model excluding households with more than two residents per bedroom had the lowest BIC (Table S2). In all reduced models, the direction of the associations for the remaining covariates was preserved (Table S3) The largest changes in coefficient magnitude occurred among socioeconomic indicators, particularly overcrowding, educational attainment, race/color composition, and municipal development. These findings suggest partial overlap among structural municipal characteristics and support the stability of the main associations, while indicating that covariate-specific effect sizes should be interpreted cautiously.

## Discussion

The study aimed to assess the spatial association of temporal dynamics, socioeconomic conditions, demographic structure, and healthcare characteristics with TB mortality across municipalities in the State of São Paulo between 2020 and 2024. The findings indicate marked spatial and temporal heterogeneity in TB mortality, associated with social vulnerability, demographic composition, healthcare access, and the territorial distribution of the TB burden.^14^^,^^23^^,^^24^

The final spatial GAMLSS model showed that higher TB incidence, household overcrowding, population ageing, and a higher proportion of individuals identifying as Black or Brown were associated with increased TB mortality, whereas higher primary healthcare coverage and better municipal socioeconomic development were associated with lower mortality. In addition, the positive temporal trend observed over the study period suggests that TB mortality remained elevated during and following the COVID-19 pandemic years.^18^ Overall, these findings indicate that TB mortality across municipalities in São Paulo is not randomly distributed across territories but rather reflects the spatial concentration of structural vulnerabilities, unequal access to healthcare, and persistent social inequities. These findings reinforce the understanding that TB mortality is shaped by structural and contextual determinants operating across both space and time.^25^

The positive association between household overcrowding and TB mortality corroborates the well-established role of precarious housing conditions in sustaining TB transmission and adverse outcomes.^24^ Municipalities with a higher proportion of households with more than two residents per bedroom exhibited higher expected TB mortality, suggesting that overcrowded living environments may contribute not only to increased exposure to *Mycobacterium tuberculosis* but also to delayed diagnosis and difficulties in treatment continuity. Previous studies have consistently demonstrated that overcrowding is associated with increased TB transmission, particularly in socially vulnerable urban settings where inadequate housing conditions coexist with poverty and limited access to healthcare.^14^^,^^26^

Similarly, municipalities with a higher proportion of older adults exhibited increased TB mortality. Ageing is an important determinant of TB severity, as older individuals are more likely to experience immunosenescence, multimorbidity, frailty, and delayed diagnosis, all of which may contribute to unfavorable outcomes. Previous investigations have demonstrated that older age groups are disproportionately affected by TB mortality and frequently experience diagnostic delays and more severe clinical presentations.^12^^,^^13^ In the context of population ageing observed in the State of São Paulo, these findings highlight the need for TB control strategies specifically adapted to older populations, including earlier detection and integrated management of chronic conditions.

The inverse association between the São Paulo Municipal Development Index and TB mortality further reinforces the role of socioeconomic development in reducing vulnerability to TB-related deaths. Municipalities with better development indicators, reflecting improved income, education, and longevity conditions, showed lower expected mortality. These findings are consistent with evidence indicating that TB remains associated with poverty and social deprivation, particularly in contexts characterized by socioeconomic inequalities.^14^^,^^26^^,^^27^

The findings evidence that the spatial heterogeneity of TB is related to territorial and socioeconomic inequalities in the state of São Paulo. Spatial analyses showed significant spatial autocorrelation among the covariates retained in the final model, indicating that TB mortality and its determinants are geographically clustered rather than randomly distributed across municipalities.

The fitted spatial effect also identified municipalities with persistently higher expected mortality even after adjustment for measured covariates, suggesting the influence of unmeasured territorial processes on TB mortality patterns. Together, these findings support previous evidence that TB mortality is spatially structured and closely associated with social vulnerability and unequal access to healthcare resources.^21^

Higher PHC coverage was associated with lower TB mortality, reinforcing the central role of PHC as the main entry point to the health system and a key component for early diagnosis, treatment follow-up, and continuity of care. PHC is essential for active case finding, directly observed treatment, contact tracing, and treatment adherence management, all of which contribute to reducing unfavorable TB outcomes.^11^^,^^28^ In territorially vulnerable settings, PHC may also mitigate the effects of social deprivation by facilitating earlier access to healthcare and continuity of care. Municipalities with lower PHC coverage may experience cumulative barriers to timely diagnosis and treatment follow-up, contributing to persistently higher TB mortality.

The inverse association observed between the proportion of adults without completed elementary education and TB mortality should be interpreted with caution. Although lower educational attainment is traditionally associated with poorer TB outcomes, the ecological nature of this study suggests that this indicator may reflect broader contextual characteristics of municipalities rather than individual educational status. Additionally, this variable may interact with other socioeconomic indicators included in the model, such as the municipal development index and PHC coverage. Therefore, this finding should not be interpreted as indicating a protective effect of lower education at the individual level, nor should causal inferences be made. Further studies using longitudinal individual-level data are needed to better elucidate this association.

The positive association between TB incidence and TB mortality was expected and indicates that municipalities with greater TB burden also experience higher mortality. This finding reinforces the close relationship between ongoing transmission, delayed diagnosis, challenges in treatment continuity, and severe disease outcomes. Previous studies have shown that areas with high TB incidence frequently concentrate social vulnerabilities and barriers to healthcare access that contribute simultaneously to disease transmission and mortality.^12^^,^^21^

The observed temporal trend showed a gradual increase in TB mortality over the study period. This finding is consistent with evidence indicating that the COVID-19 pandemic disrupted TB control activities worldwide, including reduced case detection, interruptions in treatment follow-up, reallocation of healthcare resources, and barriers to healthcare access imposed by social distancing measures.^2^^,^^18^ Importantly, the persistence of elevated mortality beyond the initial pandemic period suggests that the indirect effects of COVID-19 on TB services may have extended into subsequent years, potentially through delayed diagnosis, accumulation of untreated cases, and disruptions in continuity of care. Although municipal COVID-19 incidence was considered as a candidate covariate, it was not retained in the final model, suggesting that broader health system disruptions may have exerted a greater on TB mortality than local viral transmission intensity.

The use of GAMLSS allowed flexible modelling of the complex distributional characteristics of municipal TB mortality counts, including overdispersion, marked asymmetry, and excess zeros. Compared with conventional generalized linear models, this framework enabled a more robust representation of heterogeneous mortality patterns across municipalities, while simultaneously incorporating parametric effects, temporal structure, and residual spatial variation. The inclusion of a spatial smoothing component further improved the capacity to account for broad territorial heterogeneity not explained by measured covariates. This approach contributes methodologically to TB spatial epidemiology by integrating distributional flexibility with spatiotemporal modelling of mortality patterns.

Although this study provides important insights, several limitations should be considered. First, the ecological design and observational nature of this study do not support causal inference and may be subject to ecological fallacy. The associations identified should therefore be interpreted as relationships between contextual municipal-level characteristics and TB mortality rather than evidence of causal effects. The indicators analyzed reflect contextual municipal-level characteristics rather than direct individual exposures; therefore, observed associations should not be interpreted as individual risk relationships. Municipalities with higher proportions of vulnerable populations or unfavorable socioeconomic conditions do not necessarily imply that individuals experiencing TB mortality were directly exposed to those same conditions. In addition, some covariates were derived from census or periodically updated administrative sources and required temporal harmonization within the monthly panel structure. Although this approach allows incorporation of relevant contextual variables, it may not fully capture temporal changes occurring within municipalities during the study period and also may introduce measurement bias. He study relied on secondary mortality data, which may be subject to underreporting or misclassification, although the use of standardized ICD-10 definitions likely minimised classification bias.

The spatial component was modeled using a smooth function of municipal coordinates rather than explicit spatial dependence structures such as conditional autoregressive models. In addition, the analyses were conducted using municipal administrative boundaries, which may not fully represent the dynamic spatial organization of healthcare utilization, mobility patterns, or population interactions across territories. Consequently, spatial spillover effects may have occurred, whereby TB transmission dynamics, healthcare access, and mortality patterns extend beyond municipal borders and influence neighboring areas. However, diagnostics of residual spatial autocorrelation suggested that the adopted specification adequately captured the remaining broad spatial structure.

Overall, this study advances understanding of how demographic, socioeconomic, healthcare, and territorial determinants dynamically shape TB mortality across municipalities in São Paulo. The findings reinforce that TB mortality remains strongly associated with structural inequalities and healthcare access disparities, even in one of the most economically developed Brazilian states. Reducing TB mortality will require integrated strategies that combine strengthened PHC, improved living conditions, reduction of territorial and racial inequities, expanded social protection in highly vulnerable settings. These measures are essential not only to reduce mortality but also to support progress toward the End TB Strategy targets and reduce persistent inequities in TB outcomes across territories. This study has several strengths. First, it analyzed all registered TB deaths in the state of São Paulo over a five-year period using population-based data with municipal and monthly resolution. Second, it integrated demographic, socioeconomic, epidemiological, and healthcare indicators within a unified spatiotemporal analytical framework. Third, the use of GAMLSS allowed flexible modelling of overdispersed count data with excess zeros while simultaneously incorporating temporal and spatial components. The inclusion of a two-dimensional spatial smooth also improved the capacity to identify residual territorial heterogeneity in TB mortality patterns.

However, several limitations should be acknowledged. Some contextual variables were derived from census or administrative reference periods and treated as temporally fixed for parts of the study period. The spatial smooth captured broad residual spatial variation but did not explicitly model neighborhood dependence through formal spatial autoregressive structures. Finally, because the study relied on secondary mortality databases, underreporting or coding inconsistencies cannot be fully excluded. Despite these limitations, the consistency of the findings with previous literature and the robustness of the residual diagnostics support the validity of the proposed model. In conclusion, this study demonstrates that TB mortality in the State of São Paulo is spatially and temporally heterogeneous and strongly associated with demographic, socioeconomic, healthcare, and territorial determinants. Municipalities characterized by household overcrowding, population ageing, higher TB burden, and greater social vulnerability exhibited higher expected mortality, whereas higher primary healthcare coverage and better socioeconomic development were associated with lower mortality.

These findings reinforce that TB mortality remains closely linked to structural inequalities and unequal access to healthcare, even in one of the most economically developed states in Brazil. Strengthening primary healthcare, improving living conditions, reducing territorial and racial inequities, and implementing targeted territorial interventions are essential to reduce preventable TB deaths and support progress toward the End TB Strategy goals.

## Contributors

Conceptualization, Y.M.A, R.B.V.T, T.P.B. and R.A.A.; methodology, Y.M.A, R.B.V.T, T.P.B. and R.A.A.; formal analysis, Y.M.A, R.B.V.T, N.Z, T.P.B. and R.A.A.; investigation, Y.M.A. and R.B.V.T; data curation, Y.M.A, R.B.V.T, N.Z and R.A.A.; writing—original draft preparation, Y.M.A, R.B.V.T, N.Z, T.P.B, M.E.P.P. and R.A.A.; writing—review and editing, Y.M.A, R.B.V.T, A.CV.R, T.F.A.A, J.G.A.B, B.B.A. and J.E.F.; supervision, R.A.A.; project administration, Y.M.A; All authors have read and agreed to the published version of the manuscript. I also declare that all authors had full access to all the data in the study and had final responsibility for the decision to submit for publication. Additionally, I declare that, Y.M.A, R.B.V.T, N.Z, T.P.B. and R.A.A. have accessed and verified the data.

## Data sharing statement

The data used in this study are derived from routinely collected health information systems and are available from the corresponding author upon reasonable request, subject to applicable data protection regulations.

## Editorial disclaimer

The Lancet Group takes a neutral position with respect to territorial claims in published maps and institutional affiliations.

## Declaration of interests

The authors declare no competing interests.
