# Supplementary material for: Socioeconomic dynamics and tuberculosis mortality in São Paulo: a population-level analysis using spatial modeling
Source: Lancet Reg Health Am. 2026 Jul 13;62:101572. doi: 10.1016/j.lana.2026.101572 (PMC13382474; doi:10.1016/j.lana.2026.101572)
Supplement: Supplementary Tables [file mmc1.pdf]

SUPPLEMENTARY MATERIAL

Spatial determinants of tuberculosis mortality in São Paulo state, Brazil: a spatial GAMLSS approach

Table of Contents

Supplementary Table 1. Dimensions, indicators, definitions, sources, and reference periods of study variables .... Page 2

Supplementary Table 2. Comparison of global deviance, AIC, and BIC between the final model and sensitivity models excluding temporally assigned covariates .... Page 5

Supplementary Table 3. Coefficient estimates and p values from sensitivity models excluding temporally assigned covariates .... Page 6

Supplementary Appendix 1

GAMLSS_V2_SCRIPT……………………...…………………………………….… Page 7

Table S1. Dimensions, indicators, definitions, sources, and reference periods of study variables

Dimension	Indicator	Definition	Source	Year/ level of aggregation
**Outcome**	**Monthly TB deaths** (obitos_tb)	Monthly number of TB deaths recorded for each municipality. TB deaths were defined as deaths for which any form of TB was recorded as the underlying cause of death according to ICD-10 codes A15.0-A19.9.	Mortality Information System (SIM) [https://datasus.saude.gov.br/transferencia-de-arquivos/]	2020-2024 Monthly
**Socioeconomic**	**Sao Paulo Municipal Development Index** (ipdm)	Municipal-level development indicator composed of income, education, and longevity.	São Paulo State System for Data Analysis Foundation (SEADE) [https://dadosabertos.sp.gov.br/dataset/indice-paulista-de-desenvolvimento-municipal-ipdm]	2020 and 2022
**Socioeconomic**	**Households receiving cash transfers benefits (%)** (porcent_familia_benef_pbf_por_total_domicilio)	Proportion of households receiving cash transfer program benefits. Calculated as the number of families receiving Bolsa Família benefits divided by the total number of families in the municipality, multiplied by 100.	São Paulo State System for Data Analysis Foundation (SEADE) [https://dadosabertos.sp.gov.br/dataset/transferencia-de-rendae]	2020-2024 Monthly
**Socioeconomic**	**Adults without completed elementary education (%)** (prop_sem_fundamental_18mais)	Proportion of residents aged 18 years or older with no schooling or incomplete elementary education. Calculated as the number of residents aged 18 years or older classified as having no schooling or incomplete elementary education divided by the total number of residents aged 18 years or older, multiplied by 100.	Brazilian Institute of Geography and Statistics, 2022 Demographic Census (Accessed via public API)[https://www.ibge.gov.br/estatisticas/sociais/populacao/22827-censo-demografico-2022.html and https://sidra.ibge.gov.br/pesquisa/censo-demografico/demografico-2022/inicial]	2022
**Demographics and housing**	**Population density** (dens_demograf)	Number of inhabitants per square kilometer.	São Paulo State System for Data Analysis Foundation (SEADE) [https://dadosabertos.sp.gov.br/organization/fundacao-sistema-estadual-de-analise-de-dados-seade]	2020-2023 Annual

Dimension	Indicator	Definition	Source	Year/ level of aggregation
**Demographics and housing**	**Population aged 60 years or older (%)** (prop_mais59)	Proportion of the municipal population aged 60 years or older. Calculated as the estimated population aged 60 years or older divided by the total population, multiplied by 100.	Brazilian Institute of Geography and Statistics (Accessed via public API)[https://www.ibge.gov.br/estatisticas/sociais/populacao.html and https://sidra.ibge.gov.br/home/pmc/brasill]	2020-2024 Annual
**Demographics and housing**	**Black or brown population (%)** (prop_preta_parda)	Proportion of the municipal population self-identified as Black or Brown. Calculated as the sum of individuals self-identified as Black or Brown divided by the total population, multiplied by 100.	Brazilian Institute of Geography and Statistics, 2022 Demographic Census (Accessed via public API) [https://www.ibge.gov.br/estatisticas/sociais/populacao/22827-censo-demografico-2022.html and https://sidra.ibge.gov.br/pesquisa/censo-demografico/demografico-2022/inicial]	2022
**Demographics and housing**	**Households with more than 2 residents per bedroom (%)** (prop_mais2_dorm)	Proportion of occupied permanent private households with more than two residents per bedroom. Calculated as: households with >2 to 3 residents per bedroom plus households with >3 residents per bedroom, divided by the total number of occupied permanent private households, multiplied by 100.	Brazilian Institute of Geography and Statistics, 2022 Brazilian Demographic Census (Accessed via public API) [https://www.ibge.gov.br/estatisticas/sociais/populacao/22827-censo-demografico-2022.html and https://sidra.ibge.gov.br/pesquisa/censo-demografico/demografico-2022/inicial]	2022
**Demographics and housing**	**Households with inadequate sewage disposal (%)** (prop_esgotamento_inadequado)	Proportion of occupied permanent private households with inadequate sewage disposal. Calculated as: total occupied permanent private households minus households with adequate sewage disposal, divided by the total number of occupied permanent private households, multiplied by 100. Adequate sewage disposal included a general or stormwater sewer network, a septic tank or filter pit connected to the network, and a septic tank or filter pit not connected to the network.	Brazilian Institute of Geography and Statistics, 2022 Brazilian Demographic Census (Accessed via public API) [https://www.ibge.gov.br/estatisticas/sociais/populacao/22827-censo-demografico-2022.html and https://sidra.ibge.gov.br/pesquisa/censo-demografico/demografico-2022/inicial]	2022

Dimension	Indicator	Definition	Source	Year/ level of aggregation
**Healthcare**	**Primary health care coverage (%)**<br>(cobertura_aps)	Percentage of the municipal population potentially covered by PHC teams. Calculated using Ministry of Health parameters for eSF, eAP 20h, eAP 30h, eCR, eSFR, and eAPP teams, with the IBGE population estimate as the denominator.	Information systems of Primary Health Care (PHC) programs and services (e-GESTOR APS). According to Technical Note No. 301/2022-CGESF/DESF/SAPS/MS [https://relatorioaps.saude.gov.br/cobertura/aps]	2020-2024<br>Monthly
**Healthcare**	**Nurses per 1,000 population**<br>(taxa_enf_1000)	Rate of nurses per 1,000 inhabitants. Calculated as the total number of registered nurses divided by the total population, multiplied by 1,000.	Department of Information and Informatics of the Brazilian Unified Health System [https://tabnet.datasus.gov.br/cgi/deftohtm.exe?cnes/cnv/prid02sp.def]	2020-2024<br>Monthly
**Healthcare**	**Physicians per 1,000 population**<br>(taxa_med_1000)	Rate of physicians per 1,000 inhabitants. Calculated as the total number of registered physicians divided by the total population, multiplied by 1,000.	Department of Information and Informatics of the Brazilian Unified Health System [https://tabnet.datasus.gov.br/cgi/deftohtm.exe?cnes/cnv/prid02sp.def]	2020-2024<br>Monthly
**Epidemiological**	**Monthly TB incidence rate per 100,000 population**<br>(incid_tb_100mil)	Monthly TB incidence rate per 100,000 inhabitants. Calculated as the number of newly notified TB cases per month divided by the total population, multiplied by 100,000.	Tuberculosis Patient Monitoring System (TBweb) (The data are not publicly available and must be requested from CVE - Centro de Vigilância Epidemiológica "Prof. Alexandre Vranjac" [https://www.saude.sp.gov.br/cve-centro-de-vigilancia-epidemiologica-prof.-alexandre-vranjac/areas-de-vigilancia/tuberculose/])	2020-2024<br>Monthly
**Epidemiological**	**COVID-19 incidence rate per 100,000 population**<br>(incid_covid)	Monthly COVID-19 incidence rate per 100,000 inhabitants. Calculated as the number of newly notified COVID-19 cases per month divided by the total population, multiplied by 100,000.	Coronavirus Panel [https://covid.saude.gov.br/]	2020-2024<br>Monthly
**Epidemiological**	**Incarceration rate per 100,000 population**<br>(taxa_pen_100mil)	Rate of the population deprived of liberty per 100,000 inhabitants. Calculated as the number of people deprived of liberty in custodial institutions in the municipality divided by the total population, multiplied by 100,000.	National Penitentiary Department Information System [https://www.gov.br/senappen/pt-br/servicos/sisdepen/relatorios]	2020-2024<br>Semester

Table S2. Comparison of global deviance, AIC, and BIC between the final model and sensitivity models excluding temporally assigned covariates

**Model**	**Global deviance**	**AIC***	**BIC***
Final model	16571,64	16633,79	16899,91
Without households with >2 residents/bedroom	16575,48	16635,52	16892,62
Without adults without completed elementary education	16609,93	16675,53	16956,43
Without Black or Brown population	16580,51	16640,47	16897,21
Without São Paulo Municipal Development Index	16598,83	16662,9	16937,24

*Note: AIC = Akaike information criterion; BIC = Bayesian information criterion.

Table S3. Coefficient estimates and p values from sensitivity models excluding temporally assigned covariates

Variables	Final model	Without households with >2 residents/bedroom	Without adults without completed elementary education	Without Black or Brown population	Without São Paulo Municipal Development Index
	Estimates (p-value)	Estimates (p-value)	Estimates (p-value)	Estimates (p-value)	Estimates (p-value)
Monthly time trend	0.0048 (p<0.0001)	0.0049 (p<0.0001)	0.0064 (p<0.0001)	0.0051 (p<0.0001)	0.0062 (p<0.0001)
Households with >2 residents per bedroom (%)	0.0211 (p=0.0041)	Not included	0.0040 (p=0.57)	0.0344 (p<0.0001)	0.0381 (p<0.0001)
TB incidence rate per 100,000 population	0.0211 (p<0.0001)	0.0209 (p<0.0001)	0.0205 (p<0.0001)	0.0214 (p<0.0001)	0.0213 (p<0.0001)
Population aged 60 years or older (%)	0.0432 (p<0.0001)	0.0357 (p<0.0001)	0.0313 (p<0.0001)	0.0259 (p=0.0006)	0.0592 (p<0.0001)
Adults without completed elementary education (%)	-0.0324 (p<0.0001)	-0.0302 (p<0.0001)	Not included	-0.0315 (p<0.0001)	-0.0217 (p<0.0001)
Black or Brown population (%)	0.0142 (p<0.0001)	0.0173 (p<0.0001)	0.0120 (p=0.0001)	Not included	0.0229 (p<0.0001)
Primary health care coverage (%)	-0.0025 (p=0.0002)	-0.0025 (p=0.0002)	-0.0037 (p<0.0001)	-0.0024 (p=0.0004)	-0.0033 (p<0.0001)
São Paulo Municipal Development Index	-3.7159 (p<0.0001)	-4.0216 (p<0.0001)	-2.0064 (p<0.0001)	-4.3520 (p<0.0001)	Not included

GAMLSS_V2_SCRIPT

```
#for the model
dfgam <- c(  "time_id",  "pop_2022",  "CD_MUN7", "ano","mes",
  "ano_mes","obitos_tb",
  "incid_tb_100mil",  "dens_demograf",
  "ipdm", "porcent_familia_benef_pbf_por_total_domicilio", "taxa_pen_100mil","taxa_enf_1000",
  "taxa_med_1000",
  "prop_mais59",  "prop_preta_parda",  "cobertura_aps",
  "prop_mais2_dorm", "prop_esgotamento_inadequado",
  "prop_sem_fundamental_18mais"
)
df1 <- df1 %>%
  dplyr:: select(obitos_tb, all_of(dfgam)) %>%
  na.omit()
########## DISTRIBTUION OF OUTCOME #######
df1 %>%
  summarise(

    n = n(),
    media = mean(obitos_tb, na.rm = TRUE),
    variancia = var(obitos_tb, na.rm = TRUE),
    razao_var_media = variancia / media,
    zeros = sum(obitos_tb == 0, na.rm = TRUE),
    perc_zeros = mean(obitos_tb == 0, na.rm = TRUE) * 100, max
    = max(obitos_tb, na.rm = TRUE)
  )


#CANDIDATES PO, NBI, NBII, PIG, ZIP, ZIP2, ZINBI, ZANBI, SICHEL
m_PO <- gamlss(
  obitos_tb ~ offset(log(pop_2022)),
  data = df1,
  family = PO)

GAIC(m_PO, m_NBI, m_NBII, m_PIG, m_ZIP, m_ZIP2, m_ZINBI,m_ZANBI, m_SISCHEL, k=2)
```

```r
GAIC(m_PO, m_NBI, m_NBII, m_PIG, m_ZIP, m_ZIP2, m_ZINBI, m_ZANBI,
     m_SISCHEL, k = log(nrow(df1)))

#####
#### THREE BEST OPTIONS:
plot(m_NBI) #NBI, PIG, ZIP
wp(m_NBI)

rqres.plot(m_NBI, howmany = 10, plot.type = "all", type = "wp",
          ylim = c(-1, 1))
title(main = "Worm plot - NBI \n AIC ")
#####BEST FITTED: NBI

##############
############# TIME COMPONENT
df_tempo <- df1 %>%
  mutate(
    ano_mes = as.Date(ano_mes)) %>%
  group_by(ano_mes, ano, mes) %>%
summarise(
    obitos_tb = sum(obitos_tb, na.rm = TRUE),
    pop_total = sum(pop_2022, na.rm = TRUE),
    taxa_tb_100mil = obitos_tb / pop_total * 100000,
    .groups = "drop") %>%
  arrange(ano_mes)

############## comparing temporal components
##############
df1 <- df1 %>%
mutate(
    ano_mes = as.Date(ano_mes),
    time_id = as.integer(factor(ano_mes, levels = sort(unique(ano_mes)))),
    mes_f = factor(
    mes,
      levels = 1:12,
```

```
    labels = c("Jan", "Feb", "Mar", "Apr", "May", "Jun",
           "Jul", "Aug", "Sep", "Oct", "Nov", "Dec")))

m0 <- gamlss(
  obitos_tb ~ offset(log(pop_2022)),
  data = df1,
  family = NBI)

#options +time_id
#+ pb(time_id)
#+ mes_f
#+ pb(time_id) + mes_f

GAIC(m0, m_t_linear, m_t_smooth, m_t_season, m_t_smooth_season, k = 2)
GAIC(m0, m_t_linear, m_t_smooth, m_t_season, m_t_smooth_season, k = log(nrow(df1)))

##testando residuos temporais para ver se estão autocorrelacionados no tempo
 m0_tempo <- gamlss(
  obitos_tb ~ offset(log(pop_2022)) + time_id,
  data = df1,
  family = NBI)

df1$resid_m0_tempo <- residuals(m0_tempo)

resid_tempo_m0 <- df1 %>%
mutate(
   ano_mes = as.Date(ano_mes)) %>%
  group_by(ano_mes) %>%
  summarise(
   resid_medio = mean(resid_m0_tempo, na.rm = TRUE),
```

```
    .groups = "drop") %>%
  arrange(ano_mes)


Box.test(
  resid_tempo_m0$resid_medio,
  lag = 12,
  type = "Ljung-Box")

#############################################
########## Spatial smooth
##shapefile from IBGE
sp_sf <- st_read(
  "C:\\SP_Municipios_2025.shp",
  quiet = TRUE
) %>%
  st_make_valid() %>%
  mutate(
    CD_MUN = str_pad(as.character(CD_MUN), width = 7, pad = "0")
  ) %>%
  arrange(CD_MUN)

# factor with mun
area_levels <- sp_sf$CD_MUN


sp_sf <- sp_sf %>%
  mutate(
    area_id = factor(CD_MUN, levels = area_levels))


#mun cod in the variables df
df1 <- df1 %>%
  mutate(
    CD_MUN7 = str_pad(as.character(CD_MUN7), width = 7, pad = "0"),
    area_id = factor(CD_MUN7, levels = area_levels))
```

```
#create xy coordinates #
project shapefile
sp_proj <- st_transform(sp_sf, 5880)
# inner point of polygons
pts <- st_point_on_surface(sp_proj)
coords_mun <- pts %>%
  mutate(
    x_sp = st_coordinates(.)[, 1],

    y_sp = st_coordinates(.)[, 2]
  ) %>%
  st_drop_geometry() %>%
  dplyr::select(
    CD_MUN,
    x_sp,
    y_sp)
# merge xy with variables df
df1 <- df1 %>%
  dplyr::select(
    -any_of(c("x_sp", "y_sp"))
  ) %>%
  left_join(

  coords_mun,
    by = c("CD_MUN7" = "CD_MUN"))


#model with spatial component -------------------------------
g1 <- gamlss(
  obitos_tb ~ offset(log(pop_2022)) + time_id+
    ga(~ s(x_sp, y_sp, k=20)),
  data = df1,
  family = NBI)
##parsimonious g1 k=20
```

```
########################
######################### Moran's I
#neighborhood matrix
nb_spdep <- poly2nb(
sp_sf,
  queen = TRUE,
  row.names = area_levels) # there is 1 island

# spatial weights
listw <- nb2listw(
nb_spdep,
  style = "W",
  zero.policy = TRUE)

#######Moran's of residuals - g1 model
df1$resid_g1 <- residuals(g2)
resid_mun <- df1 %>%
  group_by(CD_MUN7) %>%
  summarise(
    resid_mean = mean(resid_g1, na.rm = TRUE),
    .groups = "drop")

map_resid <- sp_sf %>%
  st_drop_geometry() %>%
  transmute(CD_MUN7 = CD_MUN) %>%
  left_join(resid_mun, by = "CD_MUN7")

moran.test(
  map_resid$resid_mean,

  listw,
  zero.policy = TRUE,
  na.action = na.exclude)
```

```
################################ no autocorrelation
################################ I= 0.02, p= 0.183
######################
###STEP GAIC

e0 <- gamlss(
  obitos_tb ~ offset(log(pop_2022))+ time_id,
  data = df1,
  family = NBI)

e1= stepGAIC(e0, scope=list(lower = ~ offset(log(pop_2022)) + time_id,
                upper = ~ offset(log(pop_2022)) + time_id +
                dens_demograf+incid_tb_100mil+
                ipdm+porcent_familia_benef_pbf_por_total_domicilio+
                taxa_pen_100mil+taxa_enf_1000+taxa_med_1000+
                prop_mais59+prop_preta_parda+cobertura_aps+
                prop_mais2_dorm+prop_esgotamento_inadequado))
formula(e1)
#obitos_tb ~ time_id + prop_mais2_dorm + incid_tb_100mil + prop_mais59 +
#cobertura_aps + prop_preta_parda + prop_esgotamento_inadequado +
#  ipdm + offset(log(pop_2022))
summary(e1) ##################
##################

m1 <- gamlss(
  obitos_tb ~ offset(log(pop_2022)) +   time_id + prop_mais2_dorm +
    incid_tb_100mil+prop_mais59 +   prop_sem_fundamental_18mais+
    prop_preta_parda + cobertura_aps +    ipdm +
    ga(~ s(x_sp, y_sp, k = 30)),
  data = df1,
  family = NBI,
  control = gamlss.control(trace = TRUE)
```

```
)
summary(m0) #
residuals
df1$resid_m1 <- as.numeric(residuals(m1))
# mean residuals per municipality
resid_mun_m1 <- df1 %>%
  group_by(CD_MUN7) %>%
  summarise(resid_mean = mean(resid_m1, na.rm = TRUE), .groups = "drop")
  # spatial base
map_resid_m1 <- sp_sf %>%
st_drop_geometry() %>%
  transmute(CD_MUN7 = str_pad(as.character(CD_MUN), width = 7, pad = "0")) %>%
  left_join(resid_mun_m1, by = "CD_MUN7")
moran.test(
  map_resid_m1$resid_mean,

  listw,
  zero.policy = TRUE,
  na.action = na.exclude
)
#modelo COM componente espacial

moran.test(
  map_resid_m1$resid_mean,
  listw,
  zero.policy = TRUE,
  na.action = na.exclude
)
###p-value = 0.6189
###Diagnostic
rqres.plot(m1_final, howmany = 10, plot.type = "all", type = "wp",
          ylim = c(-0.3, 0.3))
title(main = "Final model")
summary(m1)
plot(m1)
```
